# Parent–child relationship quality and child psychological adjustment in families created using egg donation: children’s perspectives at age 5 years

**DOI:** 10.1093/humrep/deab265

**Published:** 2021-12-20

**Authors:** S Imrie, J Lysons, V Jadva, K Shaw, J Grimmel, S Golombok

**Affiliations:** 1 Centre for Family Research, University of Cambridge, Cambridge, UK; 2 Institute for Women’s Health, UCL, London, UK

**Keywords:** egg donation, identity-release, ART, parent–child relationship, child adjustment, Berkeley Puppet Interview

## Abstract

**STUDY QUESTION:**

What are children’s perspectives of the quality of their relationships with their parents and their own psychological well-being in families created using egg donation?

**SUMMARY ANSWER:**

Children’s scores indicated good parent–child relationship quality and high levels of psychological well-being, with children in families created using egg donation rating their relationships with their mothers as higher in warmth/enjoyment than children in a comparison group of families created using IVF.

**WHAT IS KNOWN ALREADY:**

Little is known about how children in families created through egg donation view their family relationships and their own psychological well-being. Research with 7-and-10-year-olds in anonymous egg donation families has indicated good parent–child relationship quality from children’s perspectives, but studies have not involved younger children or those conceived following identity-release egg donation.

**STUDY DESIGN, SIZE, DURATION:**

This study included 50 children who had been born through egg donation and a comparison group of 43 children conceived through IVF with the parents’ own gametes. Data were collected between April 2018 and December 2019. The sample forms part of a larger longitudinal study examining family functioning in families created through fertility treatment.

**PARTICIPANTS/MATERIALS, SETTING, METHODS:**

Children were aged 5 years old and had been born into families with different-sex couple parents. All families were visited at home. Children were administered the Berkeley Puppet Interview, a standardized assessment of parent–child relationship quality and psychological well-being.

**MAIN RESULTS AND THE ROLE OF CHANCE:**

Children in egg donation families rated their relationships with their mothers as higher in warmth and enjoyment than did children in IVF families. No differences were found between the two family types in children’s ratings of the father–child relationship, or in children’s ratings of their own psychological well-being.

**LIMITATIONS, REASONS FOR CAUTION:**

It is possible that children who did not consent to take part in the research had less positive perceptions of their family and themselves than children who participated.

**WIDER IMPLICATIONS OF THE FINDINGS:**

The findings are relevant to UK clinics offering identity-release egg donation, to parents who have used egg donation to create their family and to individuals and couples considering their fertility treatment options. That children in egg donation families were more similar than different to children in IVF families in their self-concept and perception of their family relationships should prove reassuring.

**STUDY FUNDING/COMPETING INTEREST(S):**

This research was supported by a Wellcome Trust Collaborative Award [208013/Z/17]. The authors have no conflicts of interest to declare.

**TRIAL REGISTRATION NUMBER:**

N/A.

## Introduction

In fertility treatment where a patient is not able to use their own eggs to conceive, donor eggs may sometimes be used (Lutjen *et al.*, 1984). In families formed using egg donation, the child and their gestational parent do not share a genetic connection. The number of children born as a result of assisted reproduction cycles using donor eggs continues to increase yearly. In the USA, there were over 10 000 live births from donor egg cycles started in 2018 (Centers for Disease Control and Prevention, 2021), and in the UK over 1200 babies were born following donor egg cycles in the same time period (Human Fertilisation and Embryology Authority, 2020). Little research, however, has considered family functioning in these families from the perspective of the child.

Historically, disquiet about families formed using egg donation has focused on what this lack of genetic relationship might mean for the quality of the relationship that forms between the mother and child. This concern has stemmed largely from arguments in evolutionary psychology that non-genetically related parents are less invested in their children than are genetically related ones (Daly and Wilson, 1998), and the suggestion in the adoptive parenthood literature that new adoptive parents may feel anxious about their ability to love a genetically unrelated child (Daniluk and Hurtig-Mitchell, 2003).

Nevertheless, families created using egg donation have generally been found to be functioning well, with a high quality of parenting found across studies and few differences in child psychological adjustment, parent–child relationship quality or parental psychological well-being when compared to families formed using other types of assisted reproduction or unassisted conception (see Imrie and Golombok, 2018, 2020 for reviews). Specifically, a UK longitudinal study that compared family functioning in egg donation families to sperm donation and unassisted conception families found more positive parent–child relationship quality in the gamete donation families compared to the unassisted conception families in the child’s first 3 years (Golombok *et al.*, 2004, 2005, 2006). Egg donation mothers showed more joy in their relationships with their children at age 2 years (Golombok *et al.*, 2005) and higher levels of mother–child interaction at age 3 years (Golombok *et al.*, 2006) compared to the other groups. However, poorer mother–child relationship quality was found at ages 7 and 14 years (Golombok *et al.*, 2011; Golombok *et al.*, 2017). These differences were not found on all measures, and at age 7 years were explained by families’ disclosure status (i.e. whether parent intended to tell the child about their method of conception; Golombok *et al.*, 2011). It is also worth noting that these differences were within the normal range. The parents in this longitudinal study, and indeed in the majority of research with egg donation families, had conceived their children using anonymous donation (where the donor’s identity will never be known to the family), a type of donation that is no longer permitted in some countries, including the UK, Sweden, the Netherlands and New Zealand. Much less is known about outcomes for families following the use of identity-release egg donation (i.e. where the child has the right to access identifying information about the donor on reaching adulthood), despite it potentially raising additional challenges for families around disclosure (Freeman *et al.*, 2016) and the management of boundaries between the family and donor (Imrie *et al.*, 2019).

The first phase of the current study, when children were infants (mean age* *=* *11 months), is the only study so far to have examined parent–child relationship quality in families who have used identity-release egg donation to conceive their children. The data showed no differences in father–infant relationship quality between egg donation fathers and a comparison group of IVF fathers; however, less optimal mother–child relationship quality was found in the egg donation group compared to IVF mother–infant dyads when assessed using a free-play task. Mothers through egg donation showed lower levels of sensitivity and structuring with their infants than did IVF mothers, and their infants were less optimally responsive and involving of their mothers than were IVF infants (Imrie *et al.*, 2019). It is crucial to examine whether these differences in mother–infant relationship quality continue beyond infancy and affect how children later perceive their family relationships.

The only research to date to examine parent–child relationship quality in egg donation families from children’s perspectives was part of a UK-based longitudinal study (e.g. Golombok *et al.*, 2004, 2017) in which outcomes for children in egg donation families were compared to those of children in sperm donation and unassisted conception families (Blake *et al.*, 2013). At ages 7 and 10 years, an interview assessment found no differences between family types in children’s ratings of parental warmth and affection, parents’ emotional availability, and the amount of interests/activities shared with parents, with high levels of parental warmth and involvement across all three family types. Children in egg donation families did not report a decline in shared activities with their parents between the two time points, whereas children in unassisted conception families did. The sample size in this study was relatively small though and comprised 28 egg donation children. To date, no data have been collected from children conceived using identity-release egg donation, and larger samples with younger children are needed. As parents in the UK are generally advised by fertility clinics that they should tell their children about their donor-assisted conception at an early age (Human Fertilisation and Embryology Authority, 2021), and a sizeable number of parents who used identity-release donation reported planning to tell the child before the age of 6 years (Isaksson *et al.*, 2012), it is conceivable that many children will have some knowledge of their donor conception at a younger age than those previously studied.

Traditionally within developmental psychology, young children were not considered to be accurate informants about their own experiences and problems (Edelbrock *et al.*, 1985; Boyle *et al.*, 1993). However, a consistent body of research over the last few decades has established that children from as young as 4 years old can provide reliable and informative data about multiple aspects of their lives, including their perceptions of family relationships, their psychological adjustment, their self-concept and academic ability when age-appropriate, structured instruments are used (Measelle *et al.*, 1998; Ablow, 1999; Marsh *et al.*, 2002; Clements *et al.*, 2014). For example, children’s ratings of conflict between their parents have consistently been found to predict their socio-emotional and behavioural adjustment as well as, and in some cases better than, parent-report data (Clements *et al.*, 2014). Furthermore, when examining rates of internalizing problems in children, it has been suggested that it is particularly important to collect child-report data, as it may be harder for parents and teachers to recognize internalizing problems in children than for children themselves (Achenbach *et al.*, 1987; Stone *et al.*, 2014).

One widely used and well-validated measure designed to assess children’s perceptions of themselves and their environments is the Berkeley Puppet Interview (BPI; Measelle *et al.*, 1998). This instrument, which draws on the tradition of using puppets in clinical and research applications, uses two identical puppets who engage in a peer-like exchange with the child and can be used to measure children’s perceptions of their family environment (including parent–child relationship quality and interparental conflict), their psychological adjustment, and their strengths and competencies. In terms of psychological adjustment, children’s BPI reports of their own internalizing and externalizing problems at age 6 years have been found to predict their treatment referral 2 years later (Ringoot *et al.*, 2017). Moreover, BPI data collected from children as young as 4 and 5 years of age has been found to add unique information not provided by adult-report data in relation to child conduct problems (Arseneault *et al.*, 2005) and the relation between internalizing problems and social competence (Huber *et al.*, 2019).

The current study aimed to examine parent–child relationship quality and child psychological adjustment in families created through identity-release egg donation from the perspective of children themselves at age 5 years. It forms part of the second phase of a longitudinal study examining family functioning in families created using fertility treatment (Imrie *et al.*, 2019). At Phase 1, when the children were infants, the egg donation mothers showed lower levels of sensitivity and structuring in a free-play task compared to mothers of IVF infants, and egg donation infants were also less responsive and involving of their mothers than were IVF infants (Imrie *et al.*, 2019). It is important to establish whether the less optimal mother–child relationship quality found in egg donation families continues into early childhood, when assessed from the child’s perspective. Beyond this, the study also aims to provide the first assessment of young children’s psychological adjustment in families created through egg donation, as assessed by the children themselves. The study addresses the following research questions:

- Does parent–child relationship quality differ between egg donation and IVF families, from the perspective of 5-year-old children?
- Does child psychological adjustment differ between egg donation and IVF families, from the perspective of 5-year-old children?
- What factors are associated with any differences in parent–child relationship quality and/or child psychological adjustment identified between the two family types?

## Materials and methods

### Participants

Data are presented from 50 children conceived through egg donation (*M *=* *66.84 months, SD* *=* *3.70) and a comparison group of 43 children conceived using IVF with the parents’ own gametes (*M *=* *66.79 months, SD* *=* *3.80). One target child per family was included in the study. Of the egg donation children, 43 (86%) had been conceived using identity-release donation, 7 (14%) using known donation, of which 4 (8%) used intrafamily donation. All children had been born into families with heterosexual, cisgender coupled parents, who were married or cohabiting. Nine (10%) couples had separated since Phase 1 of the study (two egg donation, seven IVF). The majority of mothers (n = 89, 96%) identified their ethnicity as White British, one (1%) mother identified as White Irish, two (2%) as White Other and one (1%) as Asian Indian. Most (n = 79, 85%) fathers identified their ethnicity as White British, one (1%) as White Irish, four (4%) as White Other, three (3%) as Asian Indian and one (1%) as Other. Ethnic identity was not known for eight fathers. Most mothers (n = 68, 74%) and fathers (n = 56, 64%) had a higher education qualification. Full demographic details can be found in Table I.

**Table I:** Family sociodemographic characteristics and fertility treatment characteristics by family type.

	Egg donation (N* *=* *50)		IVF (N* *=* *43)		Independent samples *t*-test		
	*M*	SD	*M*	SD	*t*	*p*	*d*
Child’s age (months)	66.84	3.70	66.79	3.80	0.06	0.95	0.01
Mother’s age (years)	47.58	4.24	42.33	3.93	6.16	<0.001	1.28
Father’s age (years)	47.86	5.50	44.79	5.94	2.58	0.01	0.54

	Egg donation (N* *=* *50)		IVF (N* *=* *43)		Chi-square		
	N (%)		N (%)		*χ*²	df	*p*

*Mother’s education¹*							
School education	11 (22%)		13 (30%)		0.72	1	0.40
Higher education	38 (78%)		30 (70%)				
*Father’s education*							
School education	18 (37%)		15 (35%)		0.03	1	0.85
Higher education	31 (63%)		28 (65%)				
					Fisher’s exact test		
*Multiple birth rate*							
Singletons	47 (94%)		36 (84%)				0.10
Twins	3 (6%)		7 (16%)				
					Mann–Whitney *U*-test		

*Fertility treatment*	*M*	SD	*M*	SD	*U*	*p*	*d*
No. IVF cycles to conceive child	3.56	2.38	2.02	1.21	647.00	<0.001	0.82

1 Missing data N* *=* *1 (mother), N* *=* *1 (father).

There was no difference between the groups in child age (*t*(91)* *=* *0.06, *p *=* *0.95). Egg donation mothers (*M *=* *47.58 years, SD* *=* *4.24) were significantly older than IVF (*M *=* *42.33, SD* *=* *3.93) mothers (*t*(91)* *=* *6.16, *p *<* *0.001). Egg donation fathers (*M *=* *47.86 years, SD* *=* *5.50) were significantly older than IVF (*M *=* *44.79, SD* *=* *5.94) fathers (*t*(91)* *=* *2.58, *p *=* *0.01). Egg donation couples have undergone significantly more IVF cycles to conceive their child (*t*(74.90)* *=* *4.01, *p *<* *0.001), and had fewer children in the home (*t*(91)* *=* *−3.26, *p *=* *0.002).

### Recruitment

At Phase 1, families were recruited through UK fertility clinics (see Imrie *et al.*, 2019 for full details of recruitment procedure). For the current phase of the study, families were re-contacted by the research team and asked whether they would be willing to participate in the second phase of the study. One hundred and twenty-one of the 150 families from Phase 1 participated in Phase 2, giving a retention rate of 81%.

### Procedure

Families were visited at home by two psychologists and written informed consent was obtained from a parent for the child’s participation. Verbal assent was also obtained from the child. Children were administered the BPI (Measelle *et al.*, 1998). Parents were not present during data collection with the child. Parents completed standardized questionnaires assessing their psychological well-being and relationship quality. Both phases of the study were reviewed by the University of Cambridge Psychology Research Ethics Committee. Data were collected between April 2018 and December 2019.

### Measures

#### Parent–child relationship quality and child psychological adjustment

##### Berkeley Puppet Interview

The BPI is an instrument used to obtain structured self-report data from children on multiple domains. It uses a standardized, structured and interactive interview to assess children’s perceptions of their family environment, their psychological adjustment and their strengths and competencies in an age-appropriate way. The BPI has been shown to be a reliable and valid measure (Measelle *et al.*, 1998). To administer the BPI, two identical dog puppets make opposing statements, and the child is asked to indicate which of the statements is most similar to their own experience. For example, Puppet 1: ‘I worry a lot’, Puppet 2: ‘I don’t worry a lot. How about you?’. Presenting the child with two opposing statements in a neutral tone is intended to offer either alternative as possible, and acceptable, so that the child feels comfortable enough to endorse either. The interaction involves several initial practice items to familiarize the child with the procedure. The interaction is video-recorded and later coded by trained researchers. All researchers administering and coding the measure were trained by one of the BPI authors.

For the current study, two Family Environment scales were used to assess parent–child relationship quality: Warmth and Enjoyment (six items for mother–child; six items for father–child) and Anger and Hostility (six items for mother–child; six items for father–child). Two Symptomatology scales were used to assess child psychological adjustment: Depression (seven items) and Overanxious (seven items). The Strengths and Competency scale (10 items) was also administered to assess positive functioning.

Videos were coded by one of two researchers, who did not code data that they had collected themselves. Each item is coded on a 7-point scale that captures which of the two opposing statements the child endorses and the strength of that endorsement, with a higher score indicating better functioning. Mean scores were calculated for each scale.

To establish inter-rater reliability, 24 (25.8%) randomly selected videos were coded by a second rater. Where discrepancies occurred, scores were discussed, and a final score was agreed for the purposes of analysis. The intra-class correlation coefficients for scales were as follows: mother Warmth and Enjoyment (0.94), father Warmth and Enjoyment (0.99), mother Anger and Hostility (0.94), father Anger and Hostility (0.98), Depression (0.90), Overanxious (0.90) and Strengths and Competency (0.94).

Two additional items were added to the BPI protocol in agreement with one of the authors of the measure. Adding items to the BPI protocol has been used by different researchers to introduce items specific to the topic of interest (e.g. Heberle and Carter, 2020). The first additional item comprised the following opposing statements: ‘My family is the same as other families’, ‘My family is different to other families’, with the follow-up prompt for the second statement of ‘How is your family different?’. For the second additional item, the following opposing statements were proposed to the child: ‘I’d like to change something about my family’, ‘I’d like to keep my family exactly the same’. If children endorsed the first statement the following prompt was used: ‘What would you like to change’? Responses to the first item were coded as same/different/both. Responses to the second item were coded as a dichotomous variable (same/change). Where children answered the follow-up prompts, their responses were categorized using a qualitative content analysis approach (Krippendorf, 2013) and frequencies reported.

#### Parental psychological well-being

##### Edinburgh Postnatal Depression Scale

The Edinburgh Postnatal Depression Scale (EPDS; Cox *et al.*, 1987) was administered to parents to assess depression. The EPDS has 10 items and produces a total score of 0–30. Higher scores indicate higher levels of depression. It has satisfactory split-half reliability and validity (Cox *et al.*, 1987). Cronbach’s alphas for the sample were 0.82 (mothers) and 0.87 (fathers).

##### Trait Anxiety Inventory

The Trait Anxiety Inventory (TAI; Spielberger, 1983) was used to assess parents’ anxiety levels. The 20-item questionnaire produces a total score between 20 and 80, with higher scores indicating higher levels of anxiety. The TAI has good reliability and discriminates well between non-clinical and clinical groups (Spielberger, 1983). Cronbach’s alphas were 0.88 (mothers) and 0.89 (fathers).

##### Parenting Stress Index

The short form of the Parenting Stress Index (PSI-SF; Abidin, 1990) was administered to parents to measure stress associated with parenting. The 36-item questionnaire yields total scores from 36 to 180, with higher scores reflecting greater parenting stress. The short form correlates highly with the full-length version, which has concurrent and predictive validity (Abidin, 1990). Cronbach’s alphas were 0.93 (mothers) and 0.93 (fathers).

##### Multidimensional Scale of Perceived Social Support

Parents were administered the Multidimensional Scale of Perceived Social Support (MSPSS; Zimet *et al.*, 1988) to measure their perceived social support. The questionnaire has 12 items, comprising three 4-item subscales assessing the perceived adequacy of support from friends, family and a significant other. Each item is rated on a 7-point scale, with higher scores reflecting higher perceived support. The measure has good test–retest reliability and good validity (Zimet *et al.*, 1988). Cronbach’s alphas were 0.92 (mothers) and 0.88 (fathers).

##### Golombok Rust Inventory of Marital State

Parents completed the Golombok Rust Inventory of Marital State (GRIMS; Rust *et al.*, 1990) to assess the quality of the couple relationship. Each of the 36 items is rated on a 4-point scale, with scores ranging from 0 to 84, and higher scores indicating poorer relationship quality. The GRIMS has been found to discriminate between couples who are about to separate and those who are not (Rust *et al.*, 1990). Cronbach’s alphas were 0.86 (mother) and 0.86 (fathers).

### Analysis

Differences between egg donation and IVF groups on children’s ratings for the BPI were assessed using independent samples *t*-tests. Missing data were addressed as follows: where a scale was missing data from one or two items, a mean score for the scale was calculated and the value imputed (as per the author of the measure’s guidelines). Analyses only included children who had complete data for at least one scale.

### Covariates

Egg donation and IVF groups differed in demographic (mother’s age, father’s age, number of children) and fertility treatment history (number of IVF cycles) variables. Where group differences were found for a BPI scale, correlations between the BPI scale scores of interest and other variables were conducted, and the variables that were significantly associated were controlled for using univariate analyses of covariance (ANCOVAs). This was to identify whether differences found between groups were a function of family type or could be explained by a covariate. The covariates included in the present study included demographic variables, parental psychological well-being variables and fertility treatment history variables as these are all known to be associated with parent–child relationship quality and/or child psychological adjustment (e.g. Netsi *et al.*, 2018).

Data were analysed using IBM SPSS Statistics (version 26. Armonk, NY, USA). A *p*-value equal to or <0.05 was considered to show statistical significance. Effect sizes were also calculated.

## Results

### Mother–child relationship quality

An independent samples *t*-test comparing children’s scores for the BPI scale of mothers’ warmth and enjoyment found a significant difference between egg donation and IVF families, *t*(62.27)* *=* *2.32, *p *=* *0.02. Children in egg donation families rated their mothers as showing more warmth and enjoyment in the relationship than did children in IVF families (Table II). The effect size was medium, *d *=* *0.52. There were no differences between the groups in children’s rating of their mothers’ anger and hostility, *t*(87)* *=* *−0.09, *p *=* *0.93. Mean scores indicated positive relationship quality, with both groups of children’s scores indicating that they viewed their mothers as high in warmth and enjoyment of their relationships and low in anger and hostility.

**Table II:** Results for the comparisons of Berkeley Puppet Interview scales between family types.

	Egg donation (*N *=* *50)		IVF (*N *=* *43)		Independent samples *t*-test			95% CI
	*M*	SD	*M*	SD	*t*	*p*	*d*	
Warmth and enjoyment: mother	5.94	0.35	5.70	0.55	2.32	0.02	0.52	[0.03, 0.44]
Anger and hostility: mother	5.28	0.84	5.30	0.83	−0.09	0.93	−0.02	[−0.37, 0.34]
Warmth and enjoyment: father	5.67	0.73	5.59	0.79	0.55	0.58	0.12	[−0.23, 0.41]
Anger and hostility: father	4.95	1.03	5.28	0.77	−1.68	0.10	−0.36	[−0.72, 0.06]
Depression	5.32	0.72	5.45	0.71	−0.79	0.43	−0.17	[−0.43, 0.18]
Overanxious	4.84	0.87	4.98	0.77	−0.77	0.44	−0.16	[−0.48, 0.21]
Strengths and competency	5.34	0.68	5.22	0.75	0.80	0.42	0.18	[−0.19, 0.44]

	Egg donation (N = 45)		IVF (N = 39)			Chi-square		
	*N* (%)		*N* (%)			*χ*²	*df*	*p*

*Change family*²								
Keep the same	36 (80%)		31 (82%)			0.03	1	0.86
Change	9 (20%)		7 (13%)					
*Family same/different*								
Different	35 (78%)		20 (51%)			5.99	1	0.01
Same	9 (20%)		17 (43%)					
Both	1 (2%)		2 (5%)					

2 Missing data *N *=* *1 (IVF).

Correlations between mother warmth and enjoyment and demographic, fertility treatment and parental psychological well-being variables were examined (Table III). The only variable that correlated significantly with children’s ratings of mothers’ warmth and enjoyment was fathers’ anxiety (*r *=* *0.24, *p *=* *0.05). When fathers’ anxiety was added into an ANCOVA as a covariate, the test for mothers’ warmth and enjoyment remained significant, *F*(1, 80)* *=* *5.57, *p *=* *0.02. This suggests that the group difference was a function of family type.

**Table III:** Correlations between mother warmth and enjoyment and demographic, fertility treatment and parental psychological well-being variables.

Variable	1.	2.	3.	4.	5.	6.	7.	8.	9.	10.	11.	12.	13.	14.	15.
1. Mother warmth	–														
2. Child age	0.05	–													
3. Mother age	0.07	0.09	–												
4. Father age	−0.06	0.04	0.50^***^	–											
5. No. children	−0.10	0.08	−0.17	−0.20	–										
6. No. IVF cycles	0.004	0.15	0.29^**^	0.24^*^	−0.23^*^	–									
7. PSI: mother	−0.03	−0.10	0.19	0.12	0.05	−0.23^*^	–								
8. TAI: mother	−0.07	−0.15	−0.001	0.04	0.01	−0.10	0.59^***^	–							
9. EPDS: mother	−0.10	−0.17	0.11	−0.08	0.19	−0.16	0.52^***^	0.73^***^	–						
10. GRIMS: mother	−0.08	−0.07	0.34^**^	0.30^**^	−0.14	0.14	0.56^***^	0.47^***^	0.31^**^	–					
11. MSPSS: mother	−0.10	0.09	−0.23^*^	−0.20	−0.02	−0.15	−0.39^***^	−0.38^***^	−0.37^***^	−0.44^***^	–				
12. PSI: father	0.21	−0.02	0.20	0.06	−0.05	−0.16	0.30^**^	0.10	0.14	0.10	−0.05	–			
13. TAI: father	0.24^*^	−0.02	0.12	−0.01	−0.14	0.06	0.01	−0.01	0.13	0.06	0.15	0.67^***^	–		
14. EDS: father	0.20	0.08	0.01	0.17	−0.23^*^	0.05	−0.03	−0.11	−0.04	0.002	0.10	0.48^***^	0.74^***^	–	
15. GRIMS: father	0.12	0.07	0.26^*^	0.06	−0.01	−0.23^*^	0.38^***^	0.23	0.29^*^	0.50^***^	−0.17	0.61^***^	0.41^***^	0.22	–
16. MSPSS: father	−0.05	0.06	−0.19	−0.12	−0.04	−0.09	−0.11	−0.30^**^	−0.34^**^	−0.30^*^	0.32^**^	−0.50^***^	−0.36^**^	−0.18	−0.57^***^

* *P *<* *0.05,

** *P *<* *0.01,

*** *P *< 0.001.

PSI,* *Parenting Stress Index; TAI,* * Trait Anxiety Inventory; EPDS, Edinburgh Postnatal Depression Scale; GRIMS, Golombok Rust Inventory of Marital State; MSPSS, Multidimensional Scale of Perceived Social Support.

### Father–child relationship quality

Independent samples *t*-tests comparing children’s scores on the BPI scales for fathers’ warmth and enjoyment, and anger and hostility, in the relationship were carried out. No differences were found between egg donation and IVF children’s ratings of either father warmth and enjoyment (*t*(87)* *=* *0.55, *P *=* *0.58) or anger and hostility (*t*(86)* *=* *−1.68, *p *=* *0.10. Children’s mean scores on both scales showed that in both groups, children viewed their fathers as high in warmth and enjoyment of their relationships, and low in anger and hostility.

### Psychological well-being

An independent samples *t*-test of children’s scores for depression was non-significant, *t*(85)* *=* *−0.79, *p *=* *0.43. A *t*-test of children’s scores for over-anxiousness was also non-significant, *t*(86)* *=* *−0.77, *p *=* *0.45. Thus, children in egg donation and IVF families did not differ in their ratings of their own depression and anxiety.

A *t-*test of children’s scores on the strengths and competency scale found no difference between groups in children’s ratings of their own strengths and competencies, *t*(81)* *=* *0.80, *p *=* *0.42.

Mean scores on all three scales for both groups of children were above the median, indicating that both the egg donation and IVF children had good psychological well-being.

### Thoughts about family

Eighty-four children gave a codable answer to the item asking whether they thought their family was the same or different to other families. Fifty-five (65%) children thought their family was different to other families, 26 (31%) thought their family was the same and 3 (4%) thought their family was both the same and different. Comparing only those who thought their family was the same or their family was different, children in egg donation families were more likely to say their family was different to other families than were children in IVF families, *χ*^2^(1)* *=* *5.99, *p *=* *0.01.

Of those 55 children who said that their families were different, 13 (24%) gave answers referring to their family’s physical features, 13 (24%) gave answers related to their family composition, 8 (15%) were not sure why they thought their family was different and 7 (13%) mentioned their family’s routines. Four (7%) said they had a more positive relationship than other families, three (5%) said that their family’s name was different to other families, three (5%) said that all families are different, three (5%) mentioned their family’s toys or possessions, two (4%) gave answers related to their family’s house and two (4%) children said that their family had a worse relationship than other families. Percentages do not add up to 100 as several children gave two reasons. Illustrative examples of children’s answers can be found in Table IV.

**Table IV:** Children’s (egg donation and IVF) thoughts about their family: illustrative quotations.

	*N* (%)	
*How is child’s family different to other families*		
Physical features	13 (24)	Because my daddy doesn’t have any hair and my mummy does
Family composition	13 (24)	Because we have a baby but some houses don’t
Don’t know	8 (15)	I don’t know
Family routines	7 (13)	Because we do different things… like going on holiday at different times
Better relationship	4 (7)	I do playing with my mummy and some people don’t get… some people don't get to play with their adults
Family name	3 (5)	Because my family is called [name] family
All families are different	3 (5)	If everybody would be the same then it would be weird because everyone would be like twins
Different toys	3 (5)	Because some people don’t have as much toys
House	2 (4)	Our house is different
Worse relationships	2 (4)	Because they talk to me differently. Because they do say sometimes they say I'm a stupid boy and other people don't really say that a lot to me.
*What child would change about family*		
Unsure	5 (31)	I don’t know yet
House	2 (12.5)	Build a new house. I want a farm with pigs
Family composition	2 (12.5)	Change [sister] into a boy
Family relationships	2 (12.5)	I would like to try and change their manners. I would get them to change how they speak to me. I would try and get them to stop arguing together.
Toys	1 (6)	Get more games because we’ve only got three
Everything	1 (6)	Change everything
Pet	1 (6)	I want a pet bunny
Ice-cream	1 (6)	Change ice-creams
Fantastical	1 (6)	I’d like to change my family into a cat family, I’d be a cheetah

Eighty-three children responded to the item asking whether they would keep their family the same or change something about it. Of the 83 children, 67 (81%) children said that they would keep their family the same. This did not differ according to family type, *χ*^2^(1)* *=* *0.03, *p *=* *0.86.

Of the 16 children who wanted to change something about their family, five (31%) were unsure what they would change, two (12.5%) wanted to change their house, two (12.5%) wanted to change the composition of their family and two (12.5%) wanted to improve their family’s relationships. One child wanted more toys, one child wanted to change everything about their family, one child wanted a pet, one child wanted to change the ice cream flavour the family bought and one gave a fantastical answer. Table IV shows examples of the children’s answers.

## Discussion

This study is the first to assess the quality of parent–child relationships and child psychological adjustment in families created by egg donation from the perspectives of the children themselves. Children in egg donation families viewed their relationships with their mothers as significantly higher in warmth and enjoyment than did children in the comparison group of IVF families. There were no differences between children in the two family types in their ratings of their relationships with their fathers, their levels of depression and anxiety, or their strengths and competencies.

That children in egg donation families viewed their relationships with their mothers as significantly higher in warmth than children in IVF families is particularly interesting, given that the first phase of the study found less optimal functioning in mother–infant relationship quality in egg donation dyads (Imrie *et al.*, 2019). The current finding suggests that these differences found in infancy have not affected the quality of the later mother–child relationship at age 5 years, at least from the child’s perspective. It is possible that the less optimal outcomes seen in egg donation dyads in infancy were a feature of specific challenges faced by mothers during that particular developmental phase, with qualitative research with the same sample showing that some mothers struggled with the idea of not sharing a genetic relationship with their child (Imrie *et al.*, 2020). If this does offer some explanation for the Phase 1 findings, it is also possible that non-genetic parenthood may pose less of a challenge to the mother–child relationship later in childhood when the children are 5 years, as qualitative research with other samples of parents through donor conception has found that parents feel growing confidence in their position as the child’s parent as their child grows older and the relationship develops (Kirkman, 2008; Indekeu *et al.*, 2014).

This finding of greater warmth and enjoyment in the mother–child relationship in egg donation families is in line with parent-report data from the first 3 years of a UK longitudinal study (Golombok *et al.*, 2005, 2006), and with child-report data from the same sample at age 7 and 10 years (Blake *et al.*, 2013). The group difference found in the current study could not be explained by demographic variables, families’ fertility treatment history or parental psychological well-being, and so does seem to be a function of family type. It is possible that having waited so long to have their children, egg donation mothers are especially committed to parenthood when their children do arrive (Golombok *et al.*, 2006) and that this may be reflected in their children’s perceptions of them as warmer and showing more enjoyment in the relationships.

Whereas questionnaire data collected from families with children in adolescence has indicated that mothers and children in egg donation families may have poorer quality relationships than those in sperm donation or unassisted conception families (Golombok *et al.*, 2017), the current findings suggest that this is not the case from children’s perspectives in early childhood. It is possible that egg donation becomes more challenging to the mother–child relationship during adolescence as issues related to identity become more salient for adolescents (Becht *et al.*, 2016; Golombok, 2021), but it is also important to note that the two findings may not be directly comparable. Firstly, Golombok *et al.*’s (2017) sample comprised families who had used anonymous donation, whereas the current sample had used identity-release donation, and the two donation types raise different challenges for families. Secondly, disclosure intentions, which may indirectly affect family functioning (Golombok *et al.*, 2011), differ between the two samples, with families in the current study showing much higher rates of intentions to disclose their child’s donor conception to them.

Apart from the children’s ratings of mothers’ warmth, the groups did not differ in their ratings of the parent–child relationship, with egg donation and IVF children rating their mothers and fathers as equally low in anger/hostility and the fathers as equally high in warmth. This is in line with data from egg donation families with 7-year-old children as assessed from both children’s (Blake *et al.*, 2013) and fathers’ (Casey *et al.*, 2013) perspectives. It is interesting that children in egg donation families only reported higher warmth compared to IVF children for mothers and not fathers. It is possible that given the concerns expressed by egg donation mothers in the first phase of the study about their lack of genetic connection to their child (Imrie *et al.*, 2020), mothers may consequently have felt the need to devote more time and attention to the relationship than did fathers. From a developmental systems perspective, the findings of high warmth and low hostility in parent–child relationships in egg donation families are particularly important, given that high-quality caregiver–child relationships help to establish developmental pathways that act as the foundation for later learning and social, emotional, cognitive and affective development (Osher *et al.*, 2020).

In terms of children’s psychological well-being, children in egg donation families did not differ from those in IVF families in their ratings of their own depression or anxiety, with both groups showing good psychological functioning. The only other longitudinal study of families created using egg donation has similarly found high levels of child psychological adjustment in the preschool years and middle childhood (Golombok *et al.*, 2004, 2005, 2006, 2011, 2013). The current study is the only one to have collected these data from young children themselves, and this is a particularly important addition to the existing literature as children may be better able to identify the presence of internalizing problems than parents or teachers (Stone *et al.*, 2014).

Children also did not differ in their ratings of their own strengths and competencies. Historically, studies of family functioning have conceptualized child psychological well-being as the absence of social, emotional and behavioural problems. This study moves beyond this deficits approach by also asking children to identify their strengths, a shift in focus that is reflective of a move within child mental health research towards a focus on positive development (Brownlee *et al.*, 2013).

When children were asked about whether they would change anything about their family, the vast majority said that they would keep their family the same as it is, suggesting contentment with their current situation. More children in egg donation than in IVF families thought that their family was different to other families; however, the explanations provided by children of this difference focused on ways in which their family differed in physical aspects, family structure or family routines from other families. Although children were not asked directly in this study phase about their method of conception, it is interesting that none mentioned either egg donation or IVF in their responses, despite half of the egg donation and 12% of IVF families having started the disclosure process. Children’s responses were instead consistent with the literature on children’s conceptions of social identities, which suggests that at age 5 years these conceptions are based on physical features, and at age 6 years start to develop based on group-related practices (Quintana, 1998; Bennett, 2011). It seems unlikely that at this cognitive stage, children would perceive method of conception as a type of difference, particularly given that understanding of biological inheritance does not develop until 7 years of age (Williams and Smith, 2010).

A limitation of the present study is that data were not available from all children in the full sample, so it is not possible to know whether children who took part differed in the quality of their parent–child relationships, or psychological well-being, from those who did not want to participate in the study. It is possible that children who felt less positive about their family relationships or psychological well-being may have been less willing to interact with a researcher. Furthermore, although most children responded to most items, for some BPI scales there were more than two missing items, meaning that not all children provided data for all scales. Some children withdrew consent during the BPI procedure, which accounts for the missing data as the procedure was stopped. Most often, this was because children said to the researchers that they wanted to play with something different. Nevertheless, the sample size was sufficient to detect a medium-large effect at *α* =* *0.05 (Cohen, 1992). Future studies in this area should aim to recruit larger samples in order to ensure sufficient power to detect small to medium effect sizes between groups. The current study had a retention rate of 81%, which is better than the average retention rate reported in longitudinal cohort studies (Teague *et al.*, 2018) and is in line with other longitudinal studies of assisted reproduction families with a similar follow-up period (Golombok *et al.*, 2021).

Furthermore, the BPI scales do not yield diagnoses (Ringoot *et al.*, 2013), so while they are valuable for group comparisons and it is possible to identify whether children’s scores fall in the positive or negative half of the scoring range, it is not possible to identify children experiencing clinically relevant psychiatric symptoms. Finally, the current sample is relatively homogenous in terms of parents’ educational level, ethnicity and family size and all lived in the UK. This limits the findings’ generalizability to other sociocultural contexts. To some extent, however, the sample does reflect the composition of families who have access to fertility treatment in the UK, given that most treatment is privately funded and is accessed by patients who identify their ethnicity as White British (Human Fertilisation and Embryology Authority, 2021).

Notwithstanding these limitations, the current study offers the first assessment of children’s perspectives in families created using identity-release egg donation. Although the lack of a genetic relationship between mothers and their children in egg donation families has commonly been assumed to be problematic, this does not seem to be the case when family functioning is assessed from children’s perspectives. The current findings also suggest that, despite the potential challenges posed by identity-release donation (Lampic *et al.*, 2014; Imrie *et al.*, 2019), these do not seem to have affected how 5-year-old children perceive themselves and their family relationships. Whether parents of school-aged children conceived through identity-release egg donation perceive this type of donation as affecting their family functioning is not, as yet, known and should be assessed using both quantitative and qualitative approaches. Analysis of data from the larger study from which this sample is drawn has addressed this question and these data will be reported elsewhere. Future research should also examine parent–child relationship quality and children’s psychological adjustment from other family members’ perspectives and use parental interview and parent–child observational assessments, in order to provide a more holistic assessment of family functioning.

In conclusion, the current study showed that 5-year-old children born using egg donation viewed their family relationships and themselves positively, and in a largely similar way to children born through IVF using the parents’ own gametes. These data, collected from 5-year-olds, offer a unique and little-heard perspective on family functioning in this growing new family form. The results should prove reassuring to parents who have used egg donation to create their families, and to prospective parents considering their fertility treatment options.

## Data availability

The data underlying this article cannot be shared publicly in order to maintain the privacy of individuals that participated in the study.
